# Rapid diagnosis to rule out bacterial bloodstream infection in neonates

**DOI:** 10.1186/2197-425X-3-S1-A298

**Published:** 2015-10-01

**Authors:** C Delco-Volonte, K Posfay-Barbe, O Karam

**Affiliations:** Geneva University Hospital, Geneva, Switzerland

## Introduction

Sepsis is an important cause of morbidity and mortality in neonates, especially in premature babies. Rapid and accurate diagnosis of neonatal sepsis is essential to prevent severe and life-threatening complications. However, diagnosis is often difficult as the clinical manifestations of this condition are often subtle and can overlap with those of non-infectious conditions. As a result, clinicians frequently administer empiric antibiotics to symptomatic or “at risk” infants while waiting culture results, which are currently the gold standard to identify bloodstream infection.

Prolonged and inappropriate use of antibiotics is associated with various and well documented complications and adverse events. Therefore, it is mandatory not only to rapidly treat a suspected neonatal sepsis, but also to rule out the infection as soon as possible, in order to stop unnecessary antibiotics.

## Objectives

Assess the negative predictive value of PCR/ESI-MS compared to blood cultures in neonatal sepsis.

## Methods

Prospective observational study in all consecutive neonates ( < 28 days old) with clinical suspicion of sepsis. Samples for PCR/ESI-MS analysis are collected at the same time as samples for the blood culture, before the initiation of antibiotics. The Negative Predictive Value is calculated by dividing the Real negative / (Real negative + False negative).

## Results

We have so far collected 110 samples, over the last 14 months. Eighty one of these have been analyzed so far. Two patients had negative PCR/ESI-MS results but positive blood cultures. Overall, the Negative Predictive Value is 62/64 = 97%. The 95%CI of this result is 92.6% to 100%. The final results will be presented at the Congress.

## Conclusions

Based on these preliminary results, PCR/ESI-MS analysis of neonate blood samples seems to have a very good Negative Predictive Value. This novel test might allow for early reassessment of the need for antibiotics.

